# Treatment Variation in In-Hospital Management of Out-of-Hospital Cardiac Arrest Patients: A Nationwide Retrospective Cohort Study

**DOI:** 10.7759/cureus.90986

**Published:** 2025-08-25

**Authors:** Yusuke Tsutsumi, Asuka Tsuchiya

**Affiliations:** 1 Department of Emergency Medicine, National Hospital Organization Mito Medical Center, Mito, JPN; 2 Department of Emergency and Critical Care Medicine, Tokai University School of Medicine, Isehara, JPN

**Keywords:** cpr, emergency medicine, heart arrest, out-of-hospital cardiac arrest, resuscitation

## Abstract

Background

Clinicians follow clinical practice guidelines to choose in-hospital treatment for out-of-hospital cardiac arrest (OHCA) patients. Yet, treatment practice variations may still exist for recommended interventions. In this study, we aimed to examine variations in guideline-recommended in-hospital treatments for OHCA.

Methods and findings

This nationwide cohort study utilized the Japan Association for Acute Medicine OHCA (JAAM-OHCA) registry data from 2014 to 2019. We focused on four in-hospital OHCA treatments: epinephrine administration, amiodarone administration, targeted temperature management (TTM), and coronary angiography (CAG). We included adult patients (≥18 years old) with cardiogenic etiology undergoing in-hospital resuscitation, for whom each treatment was indicated. We calculated the coefficient of variation (CV) to gauge treatment variation and used funnel plots of standardized treatment ratios to detect outlier hospitals with significantly different practices.

Results

Of the 57,754 patients in the registry, we included 26,420 in the epinephrine cohort, 1,826 in the amiodarone cohort, 6,780 in the TTM cohort, and 6,823 in the CAG cohort. Epinephrine exhibited the slightest variation (unadjusted CV 16.9%, 95% confidence interval (CI) 14.7 to 19.9; adjusted CV 15.0%, 95% CI 13.0 to 17.0). The other three in-hospital treatments had CVs ranging between 40% and 50%. Funnel plots identified outlier hospitals, accounting for six (6.6%) in the epinephrine cohort, nine (11%) in the amiodarone cohort, nine (10%) in the TTM cohort, and nine (9.9%) in the CAG cohort.

Conclusions

Epinephrine use displayed smaller practice variation than the other treatments. However, some outlier hospitals were identified even for epinephrine. This underscores the inadequacy of developing stringent guidelines and highlights the vital need for active implementation monitoring.

## Introduction

Currently, clinicians treat out-of-hospital cardiac arrest (OHCA) patients according to each country’s clinical practice guidelines, which are based on the International Liaison Committee On Resuscitation (ILCOR) guideline [1]. In Japan, the Japan Resuscitation Council (JRC) has also created guidelines based on ILCOR, and the guidelines did not differ from ILCOR in any meaningful way. The basic recommendations in each country are similar.

Nonetheless, treatment variation may exist. Previous studies suggested that the variation exists in some of the post-resuscitation treatments [2-5]. For example, several previous studies demonstrated variability in the implementation of targeted temperature management (TTM) across different hospitals [3,6,7]. Variability in the implementation of coronary angiography (CAG) has also been suggested [2,3,7]. Even for epinephrine, hospital variation in delayed epinephrine administration may exist [8]. However, treatment variation has not been fully examined for many treatments. Although these studies indicate the existence of variability, it has not been verified whether this falls within the scope of random variation. Identification and further standardization of treatments with high variability may improve prognosis.

Hence, we conducted this study to examine treatment variations in guideline-recommended in-hospital treatments for OHCA, including epinephrine, amiodarone, TTM, and CAG, using a nationwide OHCA registry in Japan. Especially, we aimed to clarify whether treatment variation exists, taking into account random variation using the funnel plot method.

## Materials and methods

We conducted a cohort study using the Japan Association for Acute Medicine OHCA (JAAM-OHCA) registry. The JAAM-OHCA is a nationwide database established by JAAM in 2014 to compile pre-hospital and in-hospital information concerning OHCA patients transported to participating hospitals. Presently, 93 hospitals have voluntarily joined to contribute data, with over 70% being tertiary care hospitals [9]. JAAM-OHCA used standardised forms to collect in-hospital information of OHCA patients. The JAAM-OHCA committee checked and confirmed the data. For prehospital information, the All-Japan Utstein Registry of the Fire and Disaster Management Agency provided the data [9]. In this study, we used the data from 2014 to 2019. The Ethics Committee of the National Hospital Organization Mito Medical Center approved this study (IRB numbers: 2024-14 and 2022-32).

We included all adult patients (≥ 18 years old) who were subjected to resuscitation attempts in the hospital. We excluded patients with confirmed non-cardiogenic etiology, as they may not be eligible for the index treatments to examine practice variations. We also excluded patients with missing information regarding the following index treatments. The other variables related to inclusion/exclusion criteria for constructing each cohort contained no missing data.

Index treatments of interest

We selected four in-hospital treatments for OHCA patients as the focus of our study: epinephrine administration during CPR, amiodarone administration for patients experiencing ventricular fibrillation (VF) or pulseless ventricular tachycardia (VT), TTM following the return of spontaneous circulation (ROSC), and CAG after ROSC as index treatments. Epinephrine (administered at a dose of 1 mg every three to five minutes during CPR) has been considered a standard treatment since the 1970s [10]. Amiodarone for VF/pulseless VT has received long-standing recommendations in guidelines, such as the 2010, 2015, and 2020 American Heart Association (AHA) guidelines, where it is classified as class 2b [11-13]. TTM and CAG have also been endorsed as post-resuscitation care in guidelines for the past decade [13-15]. In the most recent guideline, classes of recommendation were as follows: epinephrine, class 1; amiodarone, class 2b; CAG, class 1; and TTM, class 1 [16]. We selected epinephrine, TTM, and CAG because these treatments vary in previous studies [2,3,6-8]. Another reason for selecting epinephrine was that this has been recommended long time, and we aimed to clarify long-recommended treatments. We selected amiodarone because this treatment is considered controversial [17,18]; therefore, substantial treatment variation may exist.

In the cohort designed to examine epinephrine treatment variation (the epinephrine cohort), we included all eligible patients based on the specified inclusion/exclusion criteria. In the cohort aimed at examining amiodarone treatment variation (the amiodarone cohort), we further narrowed the study population to patients presenting with VF or pulseless VT upon hospital arrival. In the cohorts intended to investigate TTM and CAG treatment variation (the TTM cohort and the CAG cohort), we limited the study population to patients admitted with ROSC at the time of emergency department discharge. Furthermore, in the TTM cohort, we excluded patients admitted to a facility where TTM was not feasible. The JAAM-OHCA directly collected the information on the capability of each facility to perform TTM. These four cohorts may include overlapping patients (e.g., patients who received epinephrine, achieved ROSC, and underwent TTM would be included in both the epinephrine cohort and the TTM cohort).

Definition of the treatment variation

We define the treatment variation as the variation in the proportion of each index treatment, i.e., the number of patients who underwent each treatment divided by the number of eligible patients in each cohort, by hospital.

Statistical analyses

We assessed the proportion of each index treatment administered at each participating hospital. First, we computed the crude treatment proportion for each treatment at each participating hospital. Second, we calculated the risk-adjusted probability for each treatment at each hospital based on several a priori-defined factors. For crude treatment probability, differences in treatment probability may be based on different patient characteristics across hospitals. To adjust for this case-mix variation across hospitals, we used standardized treatment ratios. We used logistic regression models to calculate the predicted probability of treatment for each patient based on the following covariates: age, sex, initial rhythm on emergency medical services (EMS) arrival, layperson witness, bystander CPR, defibrillation by EMS personnel, prehospital epinephrine administration, prehospital airway device use, prehospital treatment by physicians, prehospital time (from EMS call to hospital arrival), and initial rhythm on hospital arrival. After excluding patients with missing index treatment information, missing data for these variables were imputed using a random-forest method within each cohort [19]. Then, we summed the predicted probabilities over the group of patients treated at each hospital to compute an expected number of treatments (E). Subsequently, we calculated the actual (observed) number of treatments (O) to derive the risk-adjusted standardized treatment ratio (O/E). Finally, the standardized treatment ratio was multiplied by the mean treatment ratio to derive the adjusted treatment proportion for each facility.

To assess the extent of variation for each treatment, we computed the coefficient of variation (CV) for both the actual (unadjusted) and adjusted treatment proportions and compared them across treatments. The CV is obtained by dividing the standard deviation (SD) by the mean and then multiplying the result by 100; it serves as an indicator for gauging the degree of variation [20]. Finally, we employed a funnel plot of the standardized treatment ratios for each treatment to investigate the presence of outlier hospitals that exhibited significant differences in their practices [21,22]. A funnel plot is widely used to compare institutional performance and detect outliers. We used a 99.8% control limit, i.e., approximately three SDs, to detect outliers. The same approach was used across all treatments with adjustment for overdispersion if necessary. All analyses were conducted using R version 4.3.1 (R Foundation for Statistical Computing, Vienna, Austria).

## Results

Patient selection

**Figure 1:**
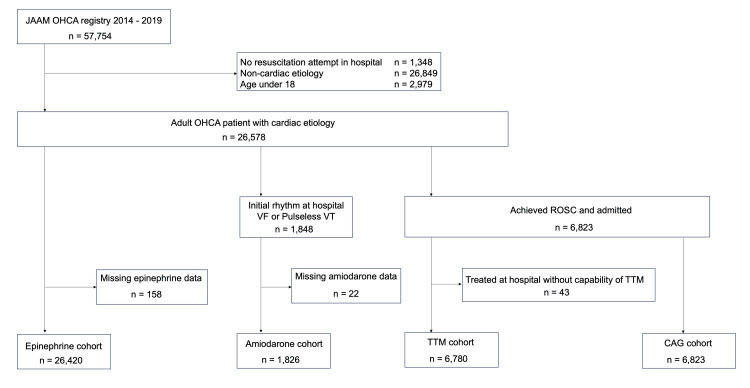
Patients flow diagram. JAAM-OHCA: Japan Association for Acute Medicine Out-of-Hospital Cardiac Arrest; VF: ventricular fibrillation; VT: ventricular tachycardia; ROSC: return of spontaneous circulation; TTM: targeted temperature management; CAG: coronary angiography

Among the 57,754 patients registered in the JAAM-OHCA between January 2014 and 2019, 26,578 were adults with a cardiac etiology. Among them, 158 patients for the epinephrine cohort, 22 patients for the amiodarone cohort, and 43 patients for the TTM cohort were excluded due to missing data. We finally included 26,420 eligible patients in the epinephrine cohort, 1,826 eligible patients in the amiodarone cohort, 6,780 eligible patients in the TTM cohort, and 6,823 eligible patients in the CAG cohort for our analyses (Figure 1).

Baseline characteristics

A total of 91, 85, 89, and 91 hospitals were included in the cohorts for epinephrine, amiodarone, TTM, and CAG, respectively. Of these, 82.1% in the epinephrine cohort, 55.0% in the amiodarone cohort, 33.3% in the TTM cohort, and 46.8% in the CAG cohort received the index treatment. The mean age of the epinephrine cohort was 74, while the mean age of the other cohort was 64 for the amiodarone cohort, 68 for the TTM cohort, and 68 for the CAG cohort. The proportion of males ranged from 62.5% in the epinephrine cohort to 82.3% in the amiodarone cohort. Across the cohorts, approximately half of patients underwent bystander CPR (48.4% to 50.3%). A total of 89.2% of the amiodarone cohort received defibrillation by EMS personnel. Approximately 60% of patients in the epinephrine cohort presented with asystole upon EMS arrival, whereas roughly 70% of patients in the amiodarone cohort exhibited VF or pulseless VT. In the three cohorts other than the epinephrine cohort, approximately 70% of patients had layperson witnesses (Table 1).

**Table 1:** Baseline characteristics of each cohort. SD: standard deviation; EMS: emergency medical systems; VF: ventricular fibrillation; VT: ventricular tachycardia; PEA: pulseless electrical activity; CPR: cardiopulmonary resuscitation; ROSC: return of spontaneous circulation * Percentages and mean (SD) were calculated excluding missing data.

	Epinephrine	Amiodarone	Targeted Temperature Management	Coronary Angiography
Index treatment	N = 26,420	N = 1,826	N = 6,780	N = 6,823
Number of hospitals	91	85	89	91
Treatment received, n (%)*	21,682 (82.1)	1,004 (55.0)	2,258 (33.3)	3,193 (46.8)
Age, mean (SD)	74 (15.0)	64 (14.8)	68 (15.7)	68 (15.7)
Sex (male), n (%)	16,523 (62.5)	1,503 (82.3)	4,872 (71.9)	4,896 (71.8)
Initial rhythm on EMS arrival, n (%)*				
VF	3,844 (14.5)	1,305 (71.5)	2,564 (37.8)	2,570 (37.7)
Pulseless VT	73 (0.3)	9 (0.5)	41 (0.6)	41 (0.6)
PEA	5,759 (21.8)	194 (10.6)	1,617 (23.8)	1,628 (23.9)
Asystole	15,531 (58.8)	235 (12.9)	1,847 (27.2)	1,870 (27.4)
Others	1,213 (4.6)	83 (4.5)	711 (10.5)	714 (10.5)
Layperson witness, n (%)	11,937 (45.2)	1,330 (72.8)	4,779 (70.5)	4,804 (70.4)
Bystander CPR, n (%)	12,793 (48.4)	919 (50.3)	3,390 (50.0)	3,407 (49.9)
Defibrillation by EMS personnel, n (%)*	5,305 (23.8)	1,584 (89.2)	3,109 (51.4)	3,120 (51.2)
Missing	4,140	50	734	734
Prehospital epinephrine administration, n (%)*	7,816 (29.6)	809 (44.3)	2,010 (29.7)	2,021 (29.6)
Missing	10	1	3	3
Prehospital airway device use, n (%)*	13,672 (62.2)	917 (60.8)	3,165 (57.3)	3,176 (57.1)
Missing	4,445	317	1,256	1,256
Prehospital treatment by physicians, n (%)*	3,239 (12.3)	390 (21.4)	1,136 (16.8)	1,136 (16.6)
Prehospital time, mean (SD)*	35 (13.2)	34 (13.7)	34 (13.7)	34 (13.7)
Missing	78	15	38	38
Initial rhythm on hospital arrival, n (%)*				
VF	1,718 (6.5)	1,706 (93.4)	1,062 (15.7)	1,069 (15.7)
Pulseless VT	122 (0.5)	120 (6.6)	61 (0.9)	61 (0.9)
PEA	5,398 (20.4)	-	1,629 (24.0)	1,640 (24.0)
Asystole	16,749 (63.4)	-	1,893 (27.9)	1,914 (28.1)
ROSC	2,433 (9.2)	-	2,135 (31.5)	2,139 (31.3)

The treatment variation

**Figure 2:**
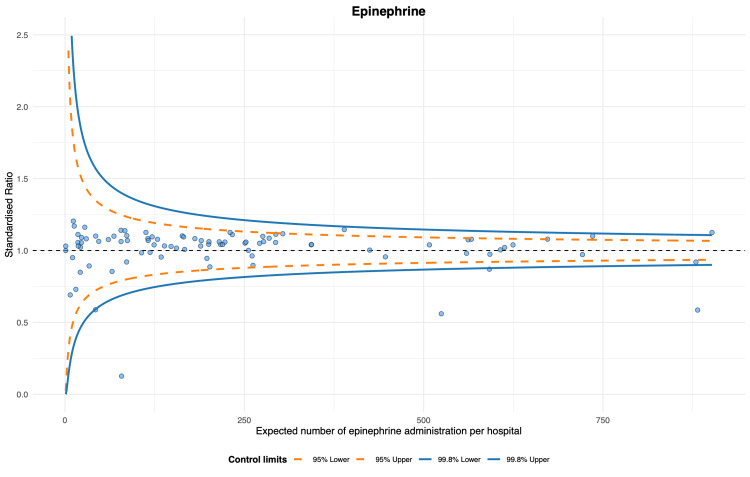
Funnel plot for epinephrine. Six hospitals were identified as outliers, with no over-dispersion detected.

**Figure 3:**
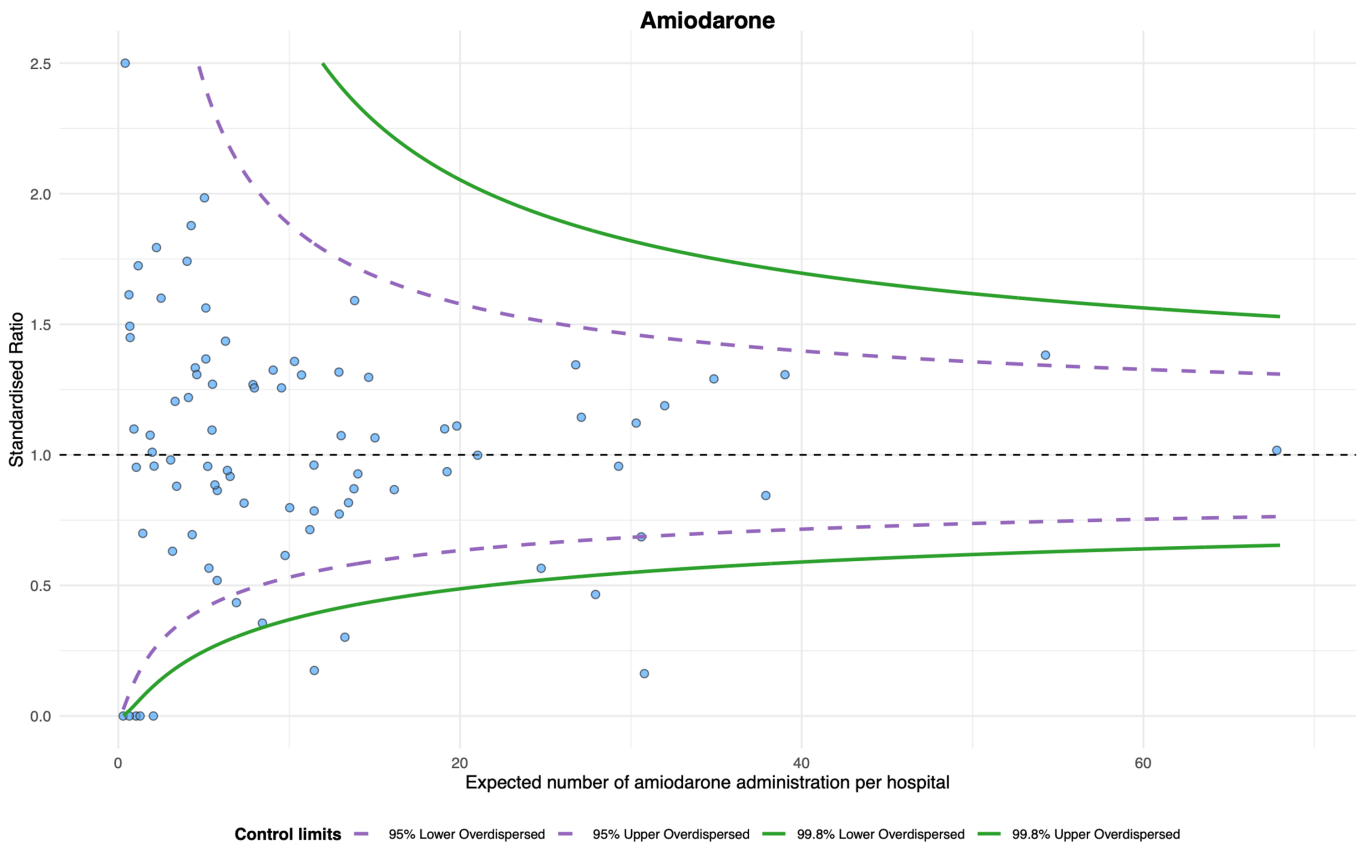
Funnel plot for amiodarone. Nine hospitals were outliers, with over-dispersion adjusted.

**Figure 4:**
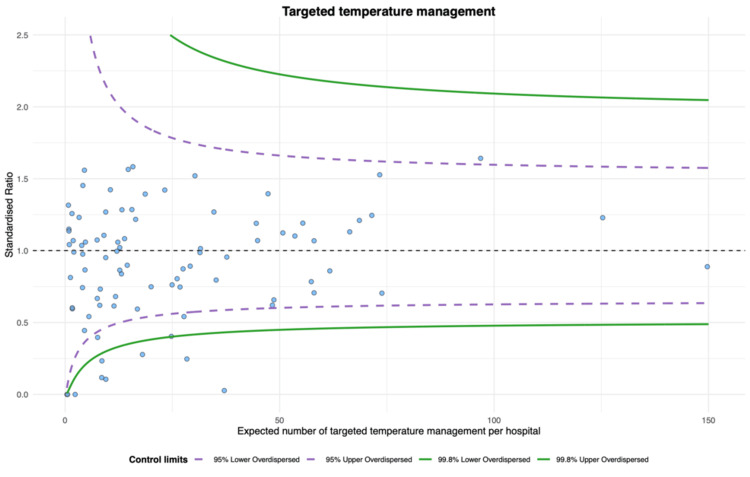
Funnel plot for targeted temperature management (TTM). Nine hospitals were outliers, with over-dispersion adjusted.

**Figure 5:**
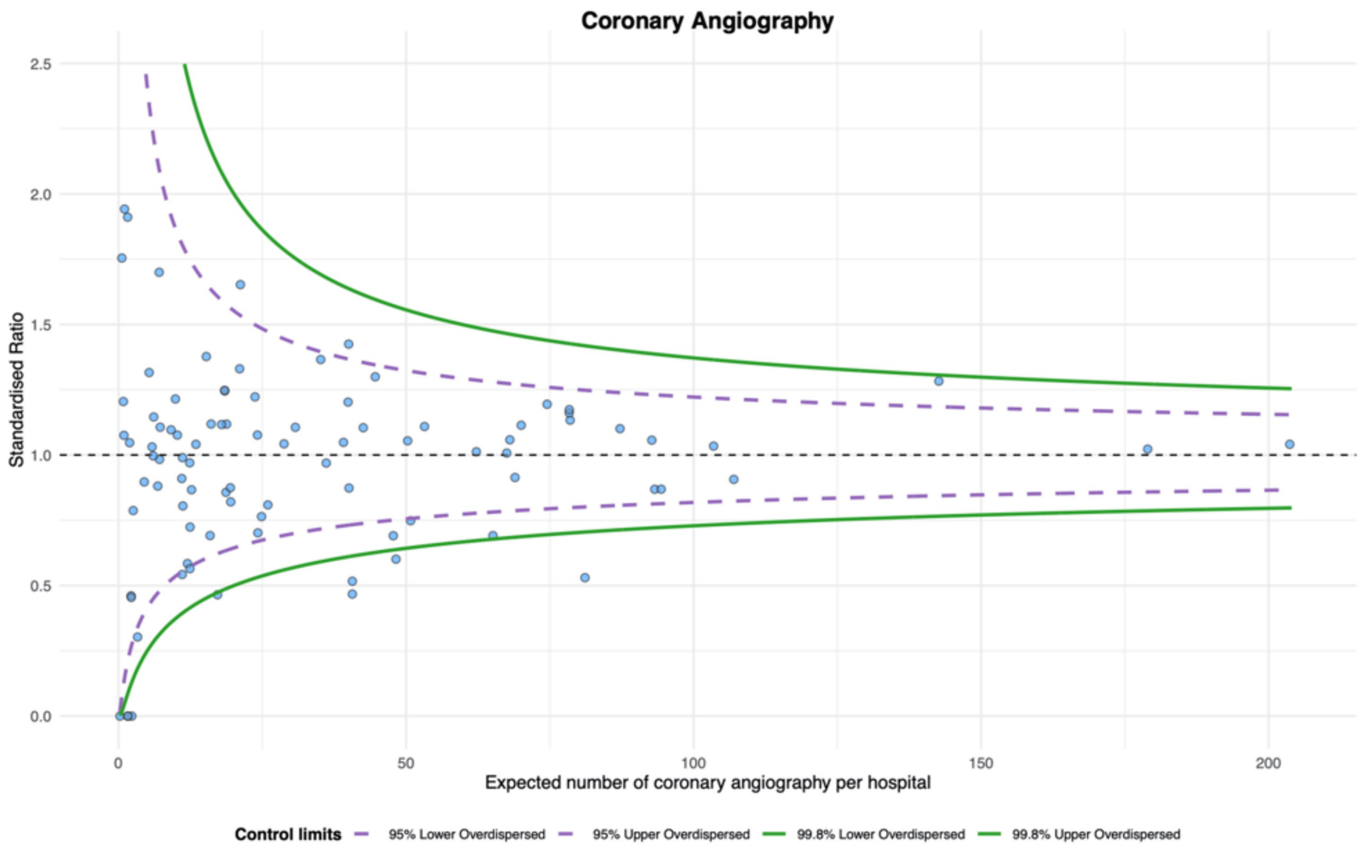
Funnel plot for coronary angiography (CAG). Nine hospitals were outliers, with over-dispersion adjusted.

The proportion of epinephrine use was the highest (unadjusted proportion: median 0.86 (IQR 0.80-0.90), adjusted proportion: median 0.86 (IQR 0.81-0.89)), while the proportion of TTM use was the lowest (unadjusted proportion: median 0.31 (IQR 0.20-0.40), adjusted proportion: median 0.33 (IQR 0.23-0.40)) (Table 2). The magnitude of variation was most minor for epinephrine (unadjusted CV, 16.9%; 95% confidence interval (CI), 14.7 to 19.9; adjusted CV, 15.0%; 95% CI, 13.0 to 17.6). The CVs for the other three index treatments were approximately 40-50% (Table 2). According to the funnel plots of standardized treatment ratios, there were six (6.6%) outlier hospitals in the epinephrine, nine (11%) in the amiodarone, nine (10%) in the TTM, and nine (9.9%) in the CAG (Figures 2-5).

**Table 2:** Treatment variation of each index treatments. IQR: interquartile range; CI: confidence interval; CV: coefficient of variation

	Epinephrine	Amiodarone	Targeted Temperature Management	Coronary Angiography
Index treatment	N = 26,420	N = 1,826	N = 6,780	N = 6,823
Number of hospitals unadjusted	91	85	89	91
Probability of undergoing the treatment, median (IQR)	0.86 (0.80-0.90)	0.57 (0.43-0.71)	0.31 (0.20-0.40)	0.43 (0.31-0.61)
CV (95% CI)	16.9 (14.7 to 19.9)	45.2 (38.4 to 55.2)	53.1 (44.9 to 65.1)	48.4 (41.1 to 58.8)
Adjusted				
Probability of undergoing the treatment, median (IQR)	0.86 (0.81-0.89)	0.56 (0.43-0.72)	0.33 (0.23-0.40)	0.48 (0.37-0.53)
CV (95% CI)	15.0 (13.0 to 17.6)	46.8 (39.7 to 57.2)	43.4 (37.1 to 52.6)	38.2 (32.8 to 45.9)
Number of outliers, n (%)	6 (6.6)	9 (11)	9 (10)	9 (9.9)

## Discussion

The results of this study revealed that among the four in-hospital treatments for OHCA, the practice variation in the use of epinephrine was notably smaller than that in the other three treatments. However, even in the case of epinephrine use, some outlier hospitals were still identified. This suggests that variations in practice may persist even in treatments with long-term recommendations.

The smaller treatment variation in epinephrine, which has been recommended for about 50 years, may suggest that epinephrine is universally used and deeply embedded in standardized advanced life support protocols, leaving little room for institutional discretion. On the other hand, the results showed considerable treatment variation in the other three treatments, even though they have been recommended for more than 10 years. This suggests that even if recommended in guidelines, it may take a relatively long period of time before the treatments become widespread and standardized in actual clinical practice. Therefore, we contend that more than merely developing guidelines may be needed, and we must ensure that treatment variations and outlier facilities are effectively addressed.

The extent of variation in TTM and CAG treatments observed in our study aligns with findings from prior research, even though the settings differed [2,3,6,23-25]. This consistency may indicate that treatment patterns for OHCA exhibit a degree of uniformity on a global scale, mainly due to the influence of national guidelines based on ILCOR recommendations. Moreover, this may be due to differences in institutional protocols, including patient selection criteria and clinician preferences. Another explanation may be due to the preferences of patients and their families, where preferences may vary depending on regional customs and ways of thinking. Additionally, we found the amiodarone cohort includes six fewer hospitals than the epinephrine cohort. This probably reflected the situation that some facilities did not have patients with initial rhythm at hospital classified as VF/pulseless VT because amiodarone is only indicated for shockable rhythms; therefore, the number of patients is inherently small (26,420 of epinephrine cohort vs. 1,826 of amiodarone cohort).

This study had several strengths. First, it is the first comprehensive examination of practice variation in guideline treatments for OHCA. Second, we utilized data from Japan, where all citizens have uniform health insurance coverage, enabling us to attribute any observed practice variation to factors other than insurance coverage. Third, the study was conducted on a nationwide scale. In addition to assessing the overall magnitude of variation, this study also identified outlier hospitals using funnel plots.

However, this study has its limitations. Firstly, owing to the inherent nature of registry data, our information is limited to what was collected. As such, we cannot entirely dismiss the possibility that outlier practices in certain facilities may have coincidentally treated patients with significantly differing conditions, such as comorbidities, arrest severity, timing, and functional status. Nonetheless, we should note that the results were analyzed using funnel plots, widely regarded as the most valid method for comparing facilities. Moreover, we adjusted for prehospital conditions and background factors based on the Utstein data, enhancing our findings' overall validity. Secondly, it's essential to recognize that this study focused primarily on practice variation and did not investigate its impact on patient outcomes. Thus, whether outlier practices significantly influenced patient outcomes remains to be seen. Thirdly, this study only examined variations based on whether or not the treatment was performed, and did not examine variations in dose or timing. Fourth, we did not assess any patient outcomes. Therefore, it remains unclear whether the treatment variation was associated with patient prognosis. Further studies are necessary to address this question. Finally, the JAAM-OHCA registry did not collect the time course of patients' rhythm change and the detailed timing of treatments. Therefore, the reasons for some results, such as patients receiving epinephrine despite ROSC, are unclear as to whether they are due to rhythm changes or misclassification during data registration.

## Conclusions

Among four in-hospital OHCA treatments, epinephrine use displayed smaller practice variation than the other treatments. However, some outlier hospitals were still identified. This study highlights the presence of practice variations, even in the treatment of OHCA, where well-established guidelines are in place. This may be due to a combination of factors, such as inadequate guideline implementation, differences in institutional protocols, including patient selection and physicians' preferences, and the preferences of patients and their families. Further investigation is needed to assess how such variability in treatment impacts patient outcomes.
